# Distance mapping and volumetric assessment of the ankle and syndesmotic joints in progressive collapsing foot deformity

**DOI:** 10.1038/s41598-023-31810-6

**Published:** 2023-03-23

**Authors:** Kevin Dibbern, Victoria Vivtcharenko, Nacime Salomao Barbachan Mansur, Matthieu Lalevée, Kepler Alencar Mendes de Carvalho, François Lintz, Alexej Barg, Andrew J. Goldberg, Cesar de Cesar Netto

**Affiliations:** 1 Department of Orthopedics and Rehabilitation, Carver College of Medicine, University of Iowa (UIOWA), 200 Hawkins Drive, Iowa City, IA 52242 USA; 2 Department of Orthopedics and Traumatology, Escola Paulista de Medicina, UNIFESP, São Paulo, Brazil; 3 Service d’orthopédie Traumatologie, Centre Hospitalier Universitaire de Rouen, Rouen, France; 4 Ramsay GDS - Clinique de L’Union, Saint-Jean, France; 5 Department of Orthopaedics, Trauma and Reconstructive Surgery, University of Hamburg, Hamburg, Germany; 6 Stanmore Royal National Orthopaedic Hospital, Stanmore, UK

**Keywords:** Musculoskeletal system, Diagnostic markers, Orthopaedics

## Abstract

The early effects of progressive collapsing foot deformity (PCFD) on the ankle and syndesmotic joints have not been three-dimensionally quantified. This case-control study focused on using weight bearing CT (WBCT) distance (DM) and coverage maps (CM) and volumetric measurements as 3D radiological markers to objectively characterize early effects of PCFD on the ankle and syndesmotic joints. Seventeen consecutive patients with symptomatic stage I flexible PCFD and 20 matched controls that underwent foot/ankle WBCT were included. Three-dimensional DM and CM of the ankle and syndesmotic joints, as well volumetric assessment of the distal tibiofibular syndesmosis was performed as possible WBCT markers of early PCFD. Measurements were compared between PCFD and controls. Significant overall reductions in syndesmotic incisura distances were observed in PCFD patients when compared to controls, with no difference in the overall syndesmotic incisura volume at 1, 3, 5 and 10 cm proximally to the ankle joint. CMs showed significantly decreased articular coverage of the anterior regions of the tibiotalar joint as well as medial/lateral ankle joint gutters in PCFD patients. This study showed syndesmotic narrowing and decreased articular coverage of the anterior aspect of the ankle gutters and talar dome in stage I PCFD patients when compared to controls. These findings are consistent with early plantarflexion of the talus within the ankle Mortise, and absence of true syndesmotic overload in early PCFD, and support DM and CM as early 3D PCFD radiological markers.

## Introduction

The assessment of the overall three-dimensional (3D) deformity in patients with Progressive Collapsing Foot Deformity (PCFD)^1,2^, previously known as *adult acquired flatfoot deformity*^3^, has evolved significantly in the last decade, mainly based on findings unveiled by Weight Bearing Computed Tomography (WBCT) imaging^4–7^. Subluxation of the foot underneath the talus, or Peritalar Subluxation (PTS)^8^, describes the hindfoot component of the deformity with the foot progressively dorsiflexing, rotating externally, and abducting under the talus. It is thought that changes resulting from PTS in PCFD can cause stresses on lateral ligamentous supports and lead to ankle involvement and arthritis^9–11^. Described markers of PTS severity in PCFD patients are the presence of sinus tarsi impingement (between the lateral process of the talus and the Gissane angle of the calcaneus, an extra articular space represented by a valley between the posterior and anterior facets), subtalar joint subluxation, and subfibular impingement (between the distal fibula and lateral aspect of the calcaneus)^12–20^. These markers were initially assessed on isolated single coronal plane WBCT images^14,15,17,21^.

Examining the impact of PTS directly on the syndesmosis, Auch and colleagues recently called the attention to significant syndesmotic widening and possible syndesmotic instability in PCFD patients secondary to chronic and progressive PTS^11^. They concluded that increased stresses in the lateral aspect of the ankle/hindfoot resulting from PTS may lead to this widening, especially in the more chronic and severe deformities. However, their study again relied on isolated single axial plane WBCT images neglecting true out-of-plane 3D assessment^14,15,17,21^.

More recently, the calculation of 3D distance maps (DM) assessing the relative position between two opposing articular surfaces (joint interaction) was validated in the foot and ankle^22,23^. Dibbern and colleagues implemented DM to evaluate intra- and extra-articular interactions between the talus and the calcaneus across the entire peritalar surface in a case-control study of PCFD patients and healthy controls^24^. In that study, they introduced the concept of coverage maps (CM), where utilizing the calculated DM between talus and calcaneus, colored maps were generated demonstrating the amount of normal interaction and coverage of the articular facets of the subtalar joint as well as abnormally increased coverage and impingement of extra-articular peritalar regions, more specifically in the sinus tarsi and subfibular areas. Later, Behrens et al. applied the same technique to the Chopart joints, demonstrating a plantarmedial coverage decrease in PCFD patients^25^.

To the authors knowledge, a similar 3D investigation of joint interaction utilizing DM to analyze the condition of the ankle and syndesmotic joints in PCFD has not been performed. Therefore, we sought to use 3D DM and CM, as well as volumetric measurements to more precisely and objectively characterize the effects of PCFD on the tibiotalar, distal tibiofibular, and ankle joint medial and lateral gutters under normal standing weightbearing conditions in early stage I (flexible) PCFD deformities. We hypothesized that compared to controls, PCFD patients would have significantly reduced anterior talar dome articular coverage due to plantarflexion of the talus, as well as increased syndesmotic volumes and distances due to chronic syndesmotic overload^11^.

## Methods

This case-control study obtained institutional review board approval (IRB #201904825), complied with the Health Insurance Portability and Accountability Act (HIPAA) and the Declaration of Helsinki. The patients signed the informed consent.

### Study design

A retrospective review of patient data collected between 2014 and 2020 was conducted to identify patients with clinical and radiographic diagnosis of PCFD who underwent a standard of care WBCT examination. Inclusion criteria for PCFD patients were age of 18 years or older, no history of prior surgical treatment in the assessed foot, complete foot, and ankle WBCT scan, flexible (PCFD Stage I) deformity, (PCFD classes A—increased hindfoot valgus; B—increased midfoot abduction; C—increased forefoot dynamic varus and/or first ray instability; and D—increased peritalar subluxation) and no valgus tilting of the ankle joint^1^. Patients with class E deformity (ankle valgus tilt/deltoid insufficiency) were not included. Control patients were selected from our WBCT imaging dataset including healthy volunteers and patients that underwent WBCT imaging for non-related acute and chronic foot and ankle pathologies, with Foot and Ankle Offset (FAO) values of normality (− 0.6 to 5.2%)^26^, and with no history of PCFD, midfoot arthritis or hallux valgus deformity (Table 1). The FAO was obtained using a dedicate software and a previously described approach^26^. After establishing the center of the foot (first metatarsal, fifth metatarsal and calcaneus) and the center of the talus, the percentage of the talar deviation from the tripod was given as the FAO^27^.

**Table 1:** Progressive collapsing foot deformity and control patients’ demographics.

Characteristic	Control (n = 20)	PCFD (n = 17)	P value
Male (No)	10	6	–
Female (No)	10	11	–
Age, mean ± SD (y)	39.1 ± 17.2	38.1 ± 17.9	0.87
BMI, mean ± SD (kg/m^2^)	33.1 ± 9.0	33.3 ± 8.7	0.98

*BMI* body mass index; *PCFD*progressive collapsing foot deformity; – no statistical comparison; SD, Standard deviation; kg, kilograms; m, meters; y, years.

The first consecutive 17 patients with symptomatic stage I flexible PCFD^1^ were selected for the study. Stage I flexible PCFD patients were selected to represent an earlier subset of deformity, allowing assessment of earlier and potentially progressive changes associated with PCFD. Twenty control patients with bilateral WBCT foot and ankle imaging were matched to have similar distributions of age, sex, and BMI (Table 1). Subjects were excluded of this group if they had any hindfoot symptoms, deformity, or arthritis in the scanned foot. WBCT scan in control patients were obtained to primarily to evaluate contralateral foot and ankle problems (with a 6-month minimal history): ankle pain (2), midfoot pain, prior distal fibular fracture (3), loose bodies in the ankle, ankle laxity, 5th metatarsal base fracture, and plantar plate injury.

### Image acquisition

WBCT studies were performed with a cone-beam CT scanner (PedCAT; Curvebeam, Pennsylvania, USA). Participants were instructed to bear weight in a natural, upright standing position with the feet approximately at shoulder width and to distribute body weight evenly between the two lower limbs.

### Volumetric measurements

**Figure 1:**
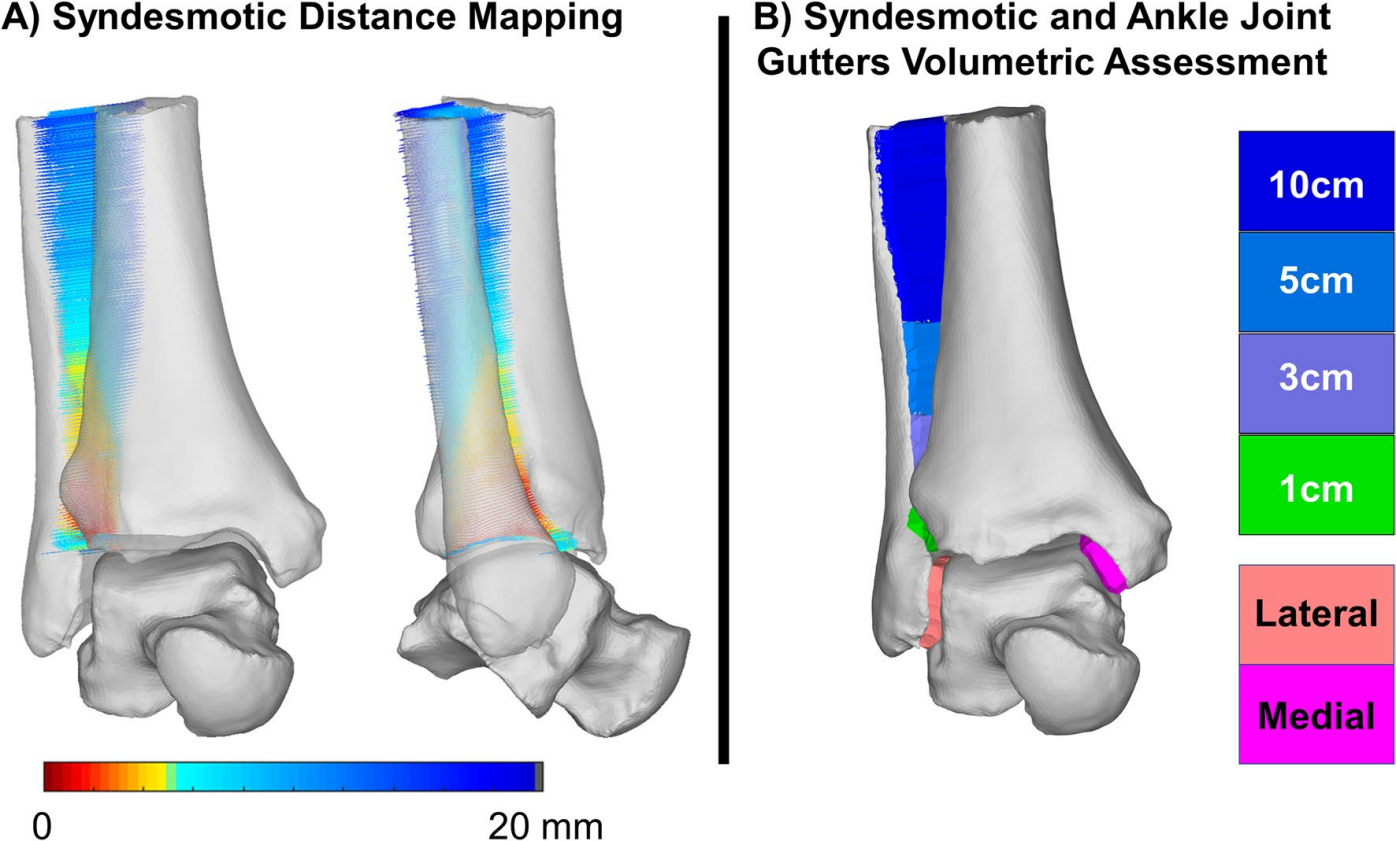
Distal tibiofibular syndesmotic joint distance mapping (**A**) and volumetric analyses (**B**). Distance maps were created (Disior Ltd, Bonelogic 1.0, Helsinki, Finland, https://www.disior.com/ortho-foot-and-ankle) along the averaged normal directions between the interfacing regions of the distal tibia and fibula (**A**). The bounded volume of these interactions (Matlab, MATLAB 2019a, MathWorks, Massachusetts, USA, https://www.mathworks.com) was automatically measured at 4 levels (1, 3, 5 and 10 cm proximally to the ankle joint) and are shown in colors from green to dark blue with the medial and lateral gutters in pink and red, respectively (**B**).

Volumetric measurements of the distal tibiofibular syndesmosis at the first 1 cm, 3 cm, 5 cm, and 10 cm from the tibiotalar joint (apex of the distal tibia articular dome) were performed, as well as the volume of the medial and lateral ankle joint gutters (medial tibiofibular and lateral talofibular articulations). Custom Matlab (MathWorks, Massachusetts, USA) code was used to automatically obtain volumes for these regions. This code first identified interfacing surfaces using the normal projections to identify opposed regions of the tibia and fibula, tibia and talus, and fibula and talus to measure volume in the syndesmosis and medial and lateral gutter, respectively. Then faces within a 30° tolerance of each other were selected to remove outliers. This defined opposed sets of points and faces on the surfaces to be enclosed to measure volume. To enclose the region between the surfaces with concave alpha shapes, points needed be filled between them at an interval less than the alpha radius. Therefore, the averaged surface normals between the bones were used to project a series of points at these intervals between the opposed surfaces. Averaged normals were used to ensure smooth boundary edges that did not include projections outside of the incisura. Points were then wrapped by a watertight concave alpha-shape using Matlab’s built in alphaShape function and an initial alpha radius of 3 mm. Bone models were compared to the alpha-shapes to ensure there were no residual convexities enclosing points inside the bone. If convexities enclosing bone existed, triangular edge subdivision was used to increase mesh density and the alpha radius and point projection were iteratively halved before being rewrapped to remove them. The 1, 3, 5, and 10 cm subdivisions of the syndesmosis were achieved by identifying the most distal point on the tibial syndesmotic interface and selecting the subset of interfacing points superiorly at those intervals using the scanners coordinate system. The resulting watertight volumes for the medial and lateral gutter and syndesmosis were then measured (Fig. 1)^24^.

### 3D distance mapping

**Figure 2:**
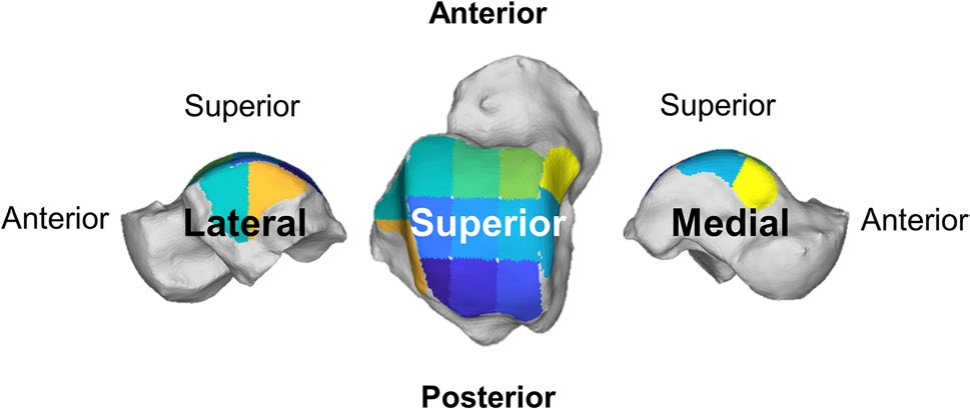
The tibiotalar joint was analyzed with respect to the talus (shown here from above) divided into a 3 × 3 grid using the principal axes of the joint surface for more detailed distance map and coverage map analyses (Disior Ltd, Bonelogic 1.0, Helsinki, Finland). The medial and lateral ankle joint gutters were also divided into anterior and posterior regions using the principal axes.

Creation of DMs began with semi-automated segmentation of the tibia, talus, and fibula using Bonelogic Ortho Foot & Ankle Software (Disior Ltd, Helsinki, Finland). Distance measurements were made based on a previously published protocol^24^, and were performed along the entire superior surface of the talus, including the tibiotalar articulation, and medial and lateral gutter articulations with the medial malleolus and the distal fibula, respectively. Distance measurements were also created in the syndesmosis for the first 1 cm, 3 cm, and 5 cm from the tibiotalar joint. For more detailed coverage analysis, the tibiotalar joint was divided into nine zones in a 3 × 3 grid using the principal axes of the joint surface while the medial and lateral gutters were divided anterior and posterior regions (Fig. 2)^25^. Measurements performed in articular areas were defined as the distance along the normal direction of vectors projected from the tibial subchondral surface to the opposed surfaces of the talus or distal fibula, as described previously^24,28^.

**Figure 3:**
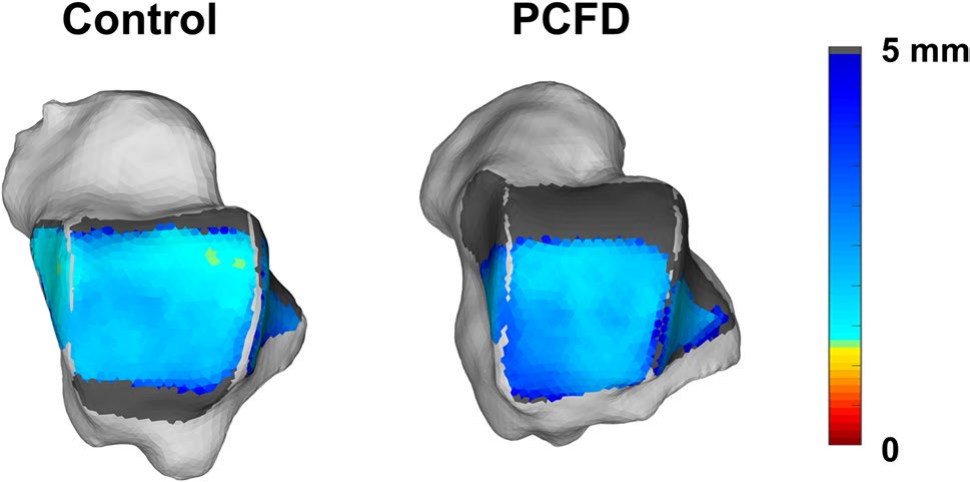
Superior view of the talus showing distance maps of the tibiotalar joint for a control and a Progressive Collapsing Foot Deformity (PCFD) patient. Dark grey regions identify areas that were not covered by the tibia (articular uncoverage).

**Figure 4:**
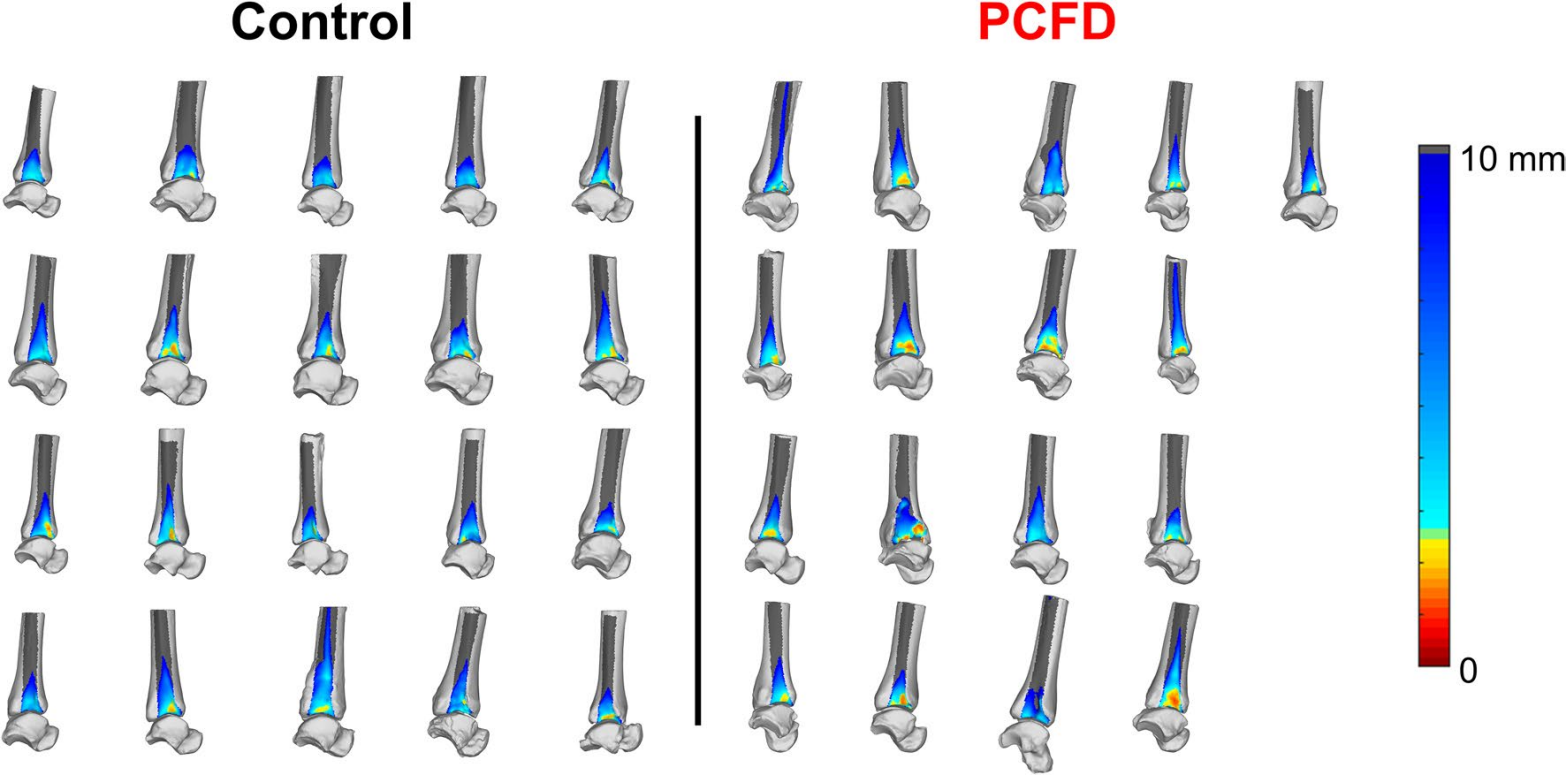
Distal tibiofibular syndesmotic colored distance maps (graded from 0 to 10 cm distances) for all control and Progressive Collapsing Foot Deformity (PCFD) patients are presented. Fibula was removed and distance maps are represented on the lateral aspect of the distal tibia. Dark grey regions identify distances > 10 mm where. Plantarflexion of the talus can be noticed on many PCFD patients.

DMs were colored to highlight regions of interest (Fig. 3). Red/Orange/Yellow colors represent close proximity and abnormal interaction, Green/Blue colors represent expected articular interactions, and gray indicates non-contacting regions or distances greater than 5 mm and 10 mm in the tibiotalar joint (Fig. 3) and tibiofibular joint (Fig. 4), respectively. Prior literature has shown that subchondral bone to bone distances rarely exceed 3 mm in the Tibiotalar articulation^23,28^.

### Coverage maps (CMs)

**Figure 5:**
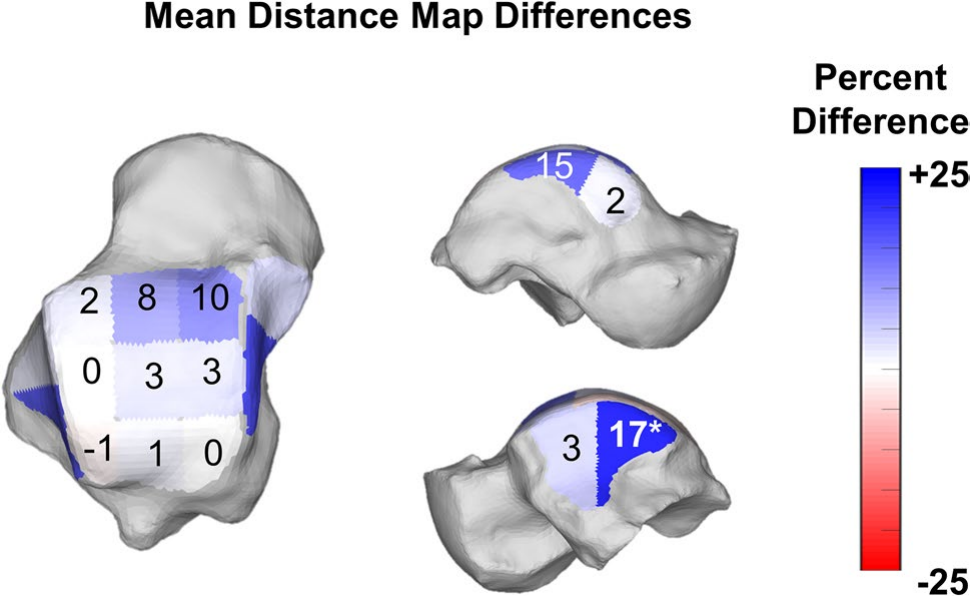
Average percent differences on Distance Maps for the nine zones of the talar dome as well as anterior and posterior aspects of the medial and lateral gutters of the ankle joint. Progressive Collapsing Foot Deformity (PCFD) minus Controls. Blue, white and red tones represent relative distances that are respectively higher, similar and smaller in PCFD patients. Bolded/starred regions denote significant changes (*p* value of the difference < .05).

Colored CMs were also created using the measured DMs and following previously published methodology^24^, aiming to better depict and highlight areas of adequate joint interaction, joint subluxation, and extra-articular impingement^25^. Pink was chosen to highlight uncoverage of articular regions that were either completely uncovered or had distances greater than 5 mm (Fig. 5). Red was chosen to indicate close bone interaction, consistent with bony impingement (if extra-articular) or arthritis (if intra-articular). For this we used DM values of less than 0.5 mm, our largest voxel size, providing an objective definition of impingement that did not rely on indirect signs like sclerosis or cysts^12^. Blue was chosen to indicate regions where joint interactions (DMs) were found to be normal, between 0.5 (no impingement) and 5 mm. The 5 mm upper threshold was chosen based on prior literature that demonstrated cartilage thickness and bone to bone distances rarely exceed 3 mm in the tibiotalar joint^22,23,28^. Finally, gray was used to indicate a shadow from the tibia and fibula in non-articular regions of the talus, where DMs were greater than 5 mm.

### Statistical analysis

Descriptive statistics of means, standard deviation, and range were reported for the DM, CM and volumetric data. Raw continuous data was initially checked for normality using the Shapiro–Wilk test. Two-tailed independent samples Student t-tests or Wilcoxon Tests were used to assess differences between control and PCFD groups, depending on the normality of their distributions. Each region in the talar dome, ankle gutters and syndesmotic space was considered independent and compared in isolation between PCFD and controls. Statistical analysis was performed by an independent observer using a dedicated software (JMP Pro, 15.0.0, SAS Institute, North Carolina, US). *P* values of 0.05 or lower were considered significant.

A post-hoc analyses power was executed using G*Power (version 3.1.9.6; Dusseldorf University, Dusseldorf, Germany). The calculation was based on WBCT syndesmosis widening values from a prior study^11^. The sample of 17 and 20 patients for each group was found to provide a 98.8% power based on mean syndesmosis distances at 1 cm and a 93.9% power based on anterior talar uncoverage.

### Ethical approval

University Ethics Committee approved this research under the number 201904825 in accordance with the Declaration of Helsinki.

### Transparency declaration

The author affirms that this manuscript is an accurate, honest, and transparent account of the study reported; that no important aspects of the study were omitted; and that any discrepancies from the study as planned (and, if relevant, registered) were carefully explained.

## Results

There were no significant differences in patient characteristics between the PCFD and control groups with respect to age (*P* = 0.87), and BMI (*P* = 0.98) distributions, though there were more males in the control group (Table 1). A summary of mean volumetric measurements for distal tibiofibular syndesmotic joint, medial, and lateral gutters are reported in Table 2.

**Table 2:** Means and Standard Deviations for Syndesmotic Volumes Measured in Millimeters Cubed, and Mean Difference in Volume from Control to Progressive Collapsing Foot Deformity Patients with P Values and 95% Confidence Intervals for Comparisons.

	Control, mm^3^		PCFD, mm^3^		MD, %	*P* value	95% CI	
	Mean	SD	Mean	SD				
Syndesmosis								
1 cm	703.0	111.7	654.9	195.7	− 6.8	0.356	− 56.3	152.5
3 cm	2611.3	522.5	2398.7	563.3	− 8.1	0.242	− 150.0	575.3
5 cm	5707.8	1422.1	5024.5	914.1	− 12.0	0.166	− 131.4	1498.0
10 cm	16,356.3	4617.7	14,137.9	2556.0	− 13.6	0.156	− 337.1	4774.0
Talar gutter								
Medial gutter	**471.7**	**178.2**	**328.9**	**97.1**	**− 30.3**	**0.012**	**44.5**	**241.1**
Lateral gutter	677.4	208.6	628.1	208.1	− 7.3	0.659	− 90.2	188.8

*PCFD* progressive collapsing foot deformity, *MD* mean difference, *SD* standard deviation, *cm* centimeter, *mm* millimeter, *CI* confidence interval.

Bolded rows denote significant changes within the region.

*Statistically significant (*p* < .05).

When compared to controls, no significant differences in the volumes measured were observed in PCFD patients across all syndesmotic regions assessed (1, 3, 5, and 10 cm proximally to the ankle joint). However, significantly smaller medial gutter volumes were detected in PCFD patients when compared to controls (*p* = 0.012), by an average of 30.3%, with no difference observed in the lateral gutter (Table 2).

When assessing syndesmotic distances (Table 3), we observed significantly smaller averaged distances measured in the syndesmotic incisura of PCFD patients at 1, 5, and 10 cm (by 17.1%, 15.4% and 19.1% and p-values of 0.002, 0.037, and 0.013, respectively). When considering averaged distances in the anterior and posterior syndesmotic regions separately, similar significantly smaller distances were seen at 1 cm (15.7% and 15.6%, *p* values 0.009 and 0.017, respectively) and 10 cm proximally to the ankle joint (13.7% and 10.7%, *p* values of respectively 0.01 and 0.043). These findings can be visualized on Fig. 4 with more yellow, orange, and red on the anterior and posterior portions of the syndesmotic incisura of PCFD when compared to controls.

**Table 3:** Means and Standard Deviations for Syndesmotic Distances Measured in Millimeters, and Mean Difference in Distances from Control to Progressive Collapsing Foot Deformity Patients with *P* Values and 95% Confidence Intervals for Comparisons.

	Control (mm)		PCFD (mm)		Mean difference (%)	*P* value	95% CI	
	Mean	SD	Mean	SD				
Syndesmosis								
1 cm	**4.78**	**0.55**	**3.96**	**0.63**	**− 17.1**	**0.002**	**0.27**	**1.04**
3 cm	5.77	0.68	5.00	0.84	− 13.3	0.058	− 0.02	1.00
5 cm	**8.20**	**1.01**	**6.94**	**0.96**	**− 15.4**	**0.037**	**0.05**	**1.50**
10 cm	**13.43**	**2.05**	**10.87**	**1.39**	**− 19.1**	**0.013**	**0.36**	**2.85**
Anterior incisura								
1 cm	**4.35**	**0.75**	**3.67**	**0.74**	**− 15.7**	**0.009**	**0.18**	**1.18**
3 cm	4.99	0.81	4.46	0.88	− 10.8	0.061	− 0.03	1.10
5 cm	6.91	1.48	6.01	1.00	− 13.0	0.057	0.04	1.75
10 cm	**11.52**	**2.49**	**9.72**	**1.43**	**− 15.6**	**0.017**	**0.41**	**3.19**
Posterior incisura								
1 cm	**5.05**	**0.80**	**4.36**	**0.72**	**− 13.7**	**0.010**	**0.18**	**1.20**
3 cm	6.19	0.96	5.70	0.94	− 7.8	0.132	− 0.15	1.12
5 cm	8.73	1.52	8.03	1.12	− 8.1	0.122	− 0.20	1.61
10 cm	**13.60**	**2.40**	**12.14**	**1.69**	** − 10.7**	**0.043**	**0.05**	**2.86**

Bolded rows denote significant changes within the region.

*PCFD* progressive collapsing foot deformity, *MD* mean difference, *SD* standard deviation, *cm* centimeter, *mm* millimeter, *CI* confidence interval.

*Statistically significant (*p* < .05).

When evaluating the mean distances in each region of the tibiotalar joint, there were no significant differences in any of the nine regions assessed (Table 4, Fig. 5). Assessment of the mean distances in the medial and lateral gutters (Table 4) found larger distances in the posterior region of the lateral gutter of PCFD patients (16.8%, *p* = 0.021), and no significant differences in the medial gutter.

**Table 4:** Means and Standard Deviations for Tibiotalar and Talofibular Articular Distances Measured in Millimeters, and Mean Difference in Distances from Control to Progressive Collapsing Foot Deformity Patients with P Values and 95% Confidence Intervals for Comparisons.

		Control (mm)		PCFD (mm)		Mean difference (%)	*P* value	95% CI	
		Mean	SD	Mean	SD				
Tibiotalar distances									
Anterior	Medial	2.80	0.41	3.09	0.55	10.3	0.078	− 0.61	0.03
	Middle	2.40	0.44	2.60	0.41	8.1	0.179	− 0.48	0.09
	Lateral	2.34	0.46	2.38	0.39	1.8	0.762	− 0.33	0.24
Middle	Medial	2.72	0.44	2.81	0.59	3.5	0.576	− 0.44	0.25
	Middle	2.55	0.49	2.64	0.53	3.5	0.598	− 0.43	0.25
	Lateral	2.60	0.45	2.60	0.46	0.0	0.796	− 0.31	0.31
Posterior	Medial	2.89	0.45	2.89	0.54	− 0.2	0.972	− 0.32	0.34
	Middle	2.77	0.45	2.80	0.52	1.2	0.837	− 0.36	0.29
	Lateral	3.10	0.49	3.08	0.53	− 0.9	0.873	− 0.31	0.37
Talar gutter distance									
Medial	Anterior	2.47	0.32	2.52	0.39	2.3	0.635	− 0.30	0.18
	Posterior	2.62	0.44	3.01	1.12	14.9	0.337	− 0.94	0.16
Lateral	Anterior	3.31	0.70	3.42	0.76	3.3	0.493	− 0.60	0.38
	Posterior	**2.82**	**0.51**	**3.29**	**0.68**	**16.8**	**0.021**	**− 0.87**	**− 0.08**

Bolded rows denote significant changes within the region.

*PCFD* progressive collapsing foot deformity, *MD*mean difference, *SD* standard deviation, *mm* millimeter, *CI* confidence interval.

*Statistically significant (*p* < .05).

**Figure 6:**
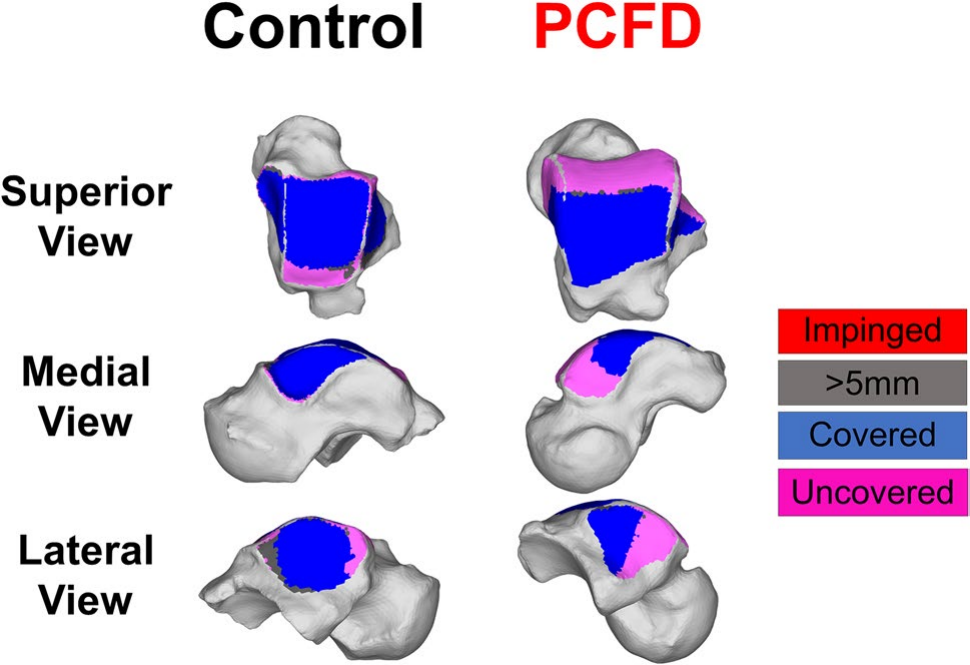
Superior, medial, and lateral views of the talus showing Coverage Maps of a Control and a Progressive Collapsing Foot Deformity (PCFD) patient. Coverage Maps identify regions of impingement (red), normal joint interaction (Blue), articular uncoverage (Pink), and interactions greater than 5 mm (Dark Grey).

**Figure 7:**
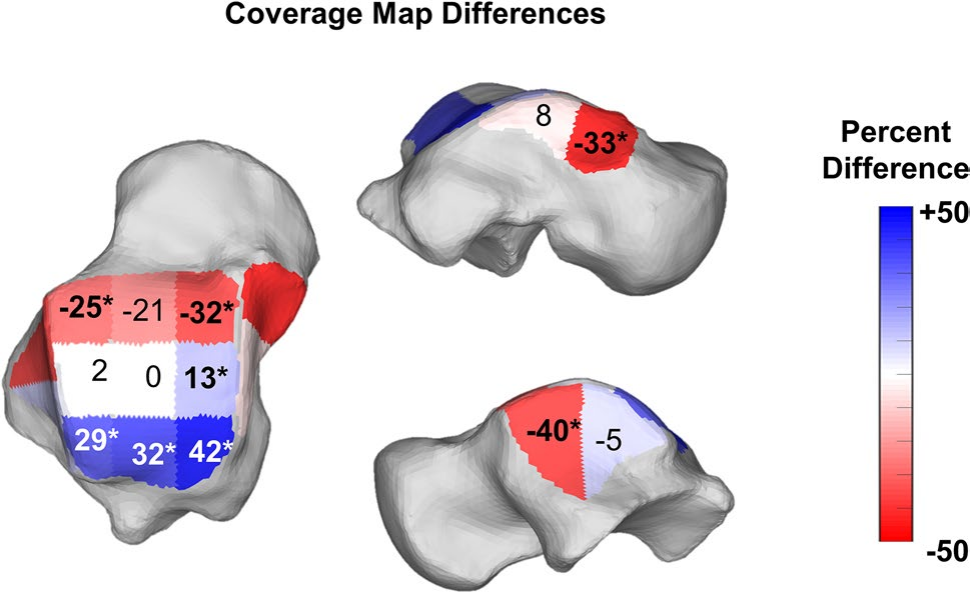
Average percent differences on Coverage Maps for the nine zones of the talar dome as well as anterior and posterior aspects of the medial and lateral gutters of the ankle joint. Progressive Collapsing Foot Deformity (PCFD) minus Controls. Blue, white and red tones represent relative distances that are respectively higher, similar and smaller in PCFD patients. Bolded/starred regions denote significant changes (*p* value of the difference < .05).

**Figure 8:**
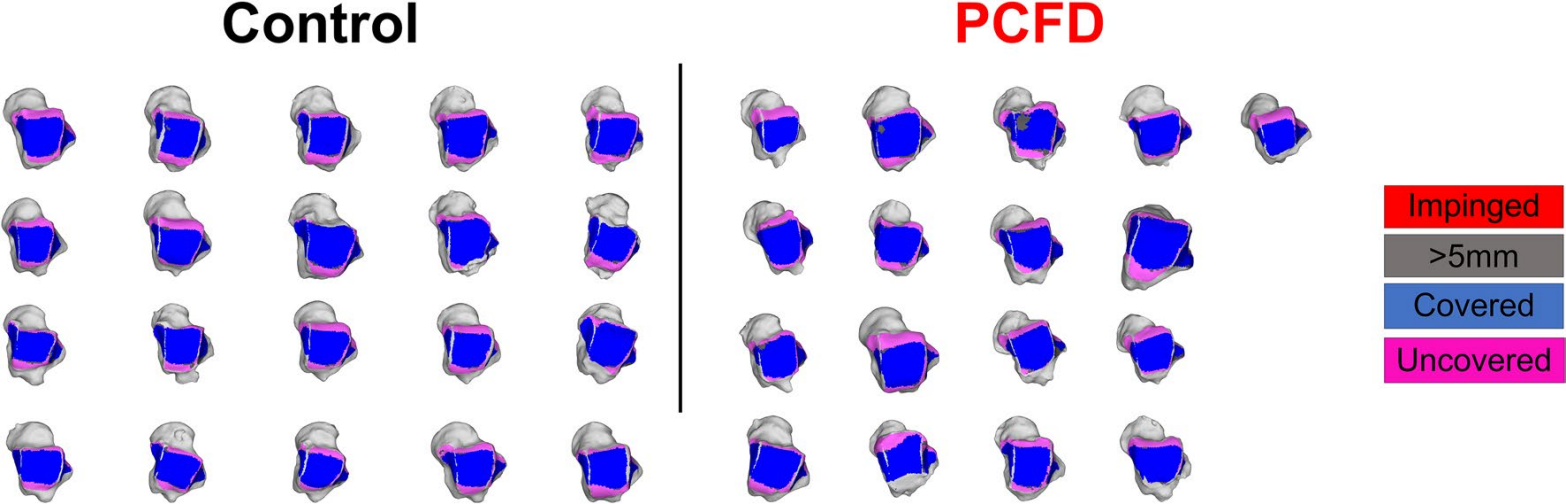
Tibiotalar joint colored Coverage Maps for all control and Progressive Collapsing Foot Deformity (PCFD) patients are presented. Tibia was removed and coverage maps are represented on the talar tome. Coverage Maps identify regions of impingement (red), normal joint interaction (Blue), articular uncoverage (Pink), and interactions greater than 5 mm (Dark Grey).

When calculating the CMs however (Figs. 6, 7, Table 5), there were significant decreases in the articular coverage of the anterior medial and anterior lateral regions of the tibiotalar joint, with mean decreases of respectively 32.3% (*p* = 0.01) and 25.2% (*p* = 0.021) in PCFD when compared to controls. Similarly, the anterior regions of both the medial and lateral gutters in the talus showed reduction in articular coverage in PCFD patients, with mean reductions of respectively 33% (*p* = 0.006) and 40.1% (*p* = 0.013). No differences were observed in the posterior aspect of the gutters. Conversely, there were significant increases in the articular coverage of all three posterior tibiotalar joint zones (average increases of 41.5%, 32.7% and 29.3%, and p-values of 0.009, 0.009, and 0.032 for respectively the medial, middle and lateral posterior zones) as well as the central medial tibiotalar zone (average increase of 13.1%, *p* = 0.034). Comparison of CMs for all PCFD and control patients are visualized in Fig. 8.

**Table 5:** Means and Standard Deviations for Tibiotalar and Talofibular Coverage Measured as a Percentage of Total Area Covered in Each Region, and Mean Difference in Coverage from Control to Progressive Collapsing Foot Deformity Patients with *P* Values and 95% Confidence Intervals for Comparisons.

		Control (%)		PCFD (%)		MD (%)	*P* value	95% CI	
		Mean	SD	Mean	SD				
Talar gutter coverage									
Medial	Anterior	**59.2**	**20.6**	**39.6**	**19.6**	** − 33.0**	**0.006**	**6.0**	**33.0**
	Posterior	75.8	14.1	81.8	14.5	8.0	0.117	− 15.6	3.5
Lateral	Anterior	**67.4**	**18.0**	**40.4**	**28.4**	** − 40.1**	**0.013**	**11.4**	**42.6**
	Posterior	97.4	3.4	92.7	8.8	− 4.8	0.097	0.4	9.0
Tibiotalar coverage									
Anterior	Medial	**59.2**	**21.1**	**40.0**	**21.2**	** − 32.3**	**0.010**	**5.0**	**33.3**
	Middle	67.5	20.8	52.8	23.5	− 21.8	0.051	− 0.1	29.5
	Lateral	**68.1**	**19.9**	**50.9**	**23.2**	** − 25.2**	**0.021**	**2.7**	**31.5**
Middle	Medial	**74.9**	**15.4**	**84.6**	**10.5**	**13.1**	**0.034**	** − 18.7**	** − 0.8**
	Middle	100.0	0.0	100.0	0.0	0.0	–	0	0
	Lateral	93.9	5.4	95.6	3.7	1.8	0.532	− 4.8	1.4
Posterior	Medial	**48.5**	**21.3**	**68.6**	**23.1**	**41.5**	**0.009**	** − 34.9**	** − 5.3**
	Middle	**58.7**	**17.8**	**77.9**	**20.0**	**32.7**	**0.009**	** − 31.8**	** − 6.6**
	Lateral	**55.7**	**23.0**	**72.0**	**21.0**	**29.3**	**0.032**	** − 31.1**	** − 1.5**

Bolded rows denote significant changes within the region.

*PCFD* progressive collapsing foot deformity, *MD* mean difference, *SD* standard deviation, *CI* confidence interval.

*Statistically significant (*p* < .05).

## Discussion

To the author’s knowledge, this is the first study to investigate the use of three-dimensional distance maps, coverage maps, and volumetric measurements of the ankle and distal tibiofibular syndesmotic joints as WBCT-based markers for PCFD. In accordance with our hypothesis, joint coverage analysis revealed significant and prominent respective decreases and increases in joint coverage of the anterior and posterior aspects of the talar dome articular surface in PCFD patients consistent with plantarflexion of the talus inside the mortise. Plantarflexion of the talus can also explain the larger averaged distances observed in the posterior aspect of the lateral gutter and decreased joint coverage in the anterior aspect of the medial and lateral gutters of the ankle joint as talar plantarflexion places the narrower posterior talus in articulation with the distal fibula and medial malleolus^29,30^. This talar malpositioning is a known phenomenon in the pathogenesis of PCFD, a true manifestation of PTS^14,31^. As the foot moves in external rotation under and distal to the talus, the talar bone assumes a internal rotation and plantarflexed position^30,32,33^. The talar head becomes uncovered and some of the collapse is established^25^. Reduced outward forces from the talus on the syndesmosis in this position can also be expected potentially explaining the decreased syndesmotic distances that contrasted with our hypothesis and previous findings of widening. These changes were observed despite a notable absence of any decreases in tibiotalar joint distance measures across all regions in the articulation.

Volumetric syndesmotic incisura measurements have been shown to be accurate in diagnosing traumatic syndesmotic instability^34,35^. Syndesmotic widening has also been recently demonstrated in PCFD patients in a case-control study with 62 symptomatic PCFD and 29 controls where the authors measured the distal tibiofibular syndesmotic incisura area in a single axial WBCT image, 1 cm proximally to the ankle joint^11^. An explanation for syndesmotic widening and possible syndesmotic instability in PCFD patients would be related to persistent and progressively increased stresses in the lateral aspect of the tibiotalar, talofibular and subtalar joints secondary to PTS, sinus tarsi and subfibular impingements, as well as possible valgus tilting of the talus under the mortise^12,18,36–39^. The same authors demonstrated that although there was syndesmotic widening in PCFD patients, the correlation between deformity severity, measured by the Foot and Ankle Offset (FAO), and syndesmotic widening was weak^11^. They also demonstrated that the widening was more pronounced when FAO values were between 7 and 9.3%, a range that can be considered as moderate to severe collapse and hindfoot valgus in PCFD patients, since values of FAO in healthy non-PCFD patients being reported as normal when up to 5.2%^26^. The assessments performed in our study should be considered as a more complete and 3D evaluation when compared to Auch et al.^11^ since this study measured distance and volumes throughout the entire imaged extension of the distal tibiofibular syndesmosis, up to 10 cm proximal to the ankle joint, rather than a 2D area assessment performed in a single axial plane image. In our assessment, we did not confirm the initially expected syndesmotic widening observed by previous authors. Conversely, we identified that overall syndesmotic distances were smaller in PCFD patients when compared to controls. A possible explanation for this could be that as the talus plantarflexes inside the mortise, likely secondarily to a loss of bony and ligamentous support related to the PTS, the thinner posterior part of the talus moves to interface with the mortise, explaining the increased averaged distances in the posterior aspect of the medial and lateral gutters of the ankle joint. This same talar plantarflexion motion would also allow the distal fibula to rotate internally and displace medially^40–42^, decreasing width of the mortise and potentially explaining the overall smaller averaged syndesmotic incisura distances observed in PCFD patients when compared to controls. Another possible explanation for decreased syndesmotic distances in our cohort of PCFD patients would be that our cohort, composed of patients with early and less pronounced deformity, was not severe enough to be consistent with chronic lateral impingement and increased syndesmotic overload. In that hypothetical scenario, the resultant effects associated with talar plantarflexion could occur earlier, bringing the narrower part of the talar dome into the mortise, unloading the syndesmosis, allowing distal fibular internal rotation and subsequent decrease in the overall distances between the distal tibia and fibula. The anterior talofibular ligament could exaggerate this movement pulling the fibula along with the talus into internal rotation. Taken together, our results suggest that syndesmotic widening may be a symptom of more severe later stage PCFD, occurring secondary to chronic lateral stresses associated with sinus tarsi and primarily subfibular impingement^18^. It also suggests that subfibular narrowing/impingement may provide an early marker for subsequent progression to increased syndesmotic overload, widening and instability. However, additional research is needed to confirm these possibilities.

As previously mentioned, the distance maps of all nine talar dome zones did not show any differences between PCFD and control patients. This indicates that our PCFD cohort had not yet developed any important arthritic changes (narrowing) or valgus talar tilting (widening) in the tibiotalar joint. It is well accepted that these are usually late findings in more severe PCFD^43,44^. However, the 3D CMs assessment of relative positioning between talus and distal tibia revealed significantly increased joint uncoverage in the anterior aspect of the talus and increased coverage of the posterior talar dome. These findings can be explained by increased plantarflexion and possible slight anterior translation of the talus relative to the tibia in patients with PCFD^36,45,46^. Our hope and understanding is that 3D DM assessment in PCFD can serve as a better WBCT-based radiologic marker for initial and subtle deformities in PCFD when compared to conventional angular assessments of severity of longitudinal medial arch collapse and talus plantarflexion, such as of talar position relative to the distal tibia, calcaneus, and first metatarsal. PCFD measurements that include assessment of the longitudinal axis of the talus have been shown to not to have high reliability and reproducibility due to challenges and inconsistency in reproducing accurate measurements^47^; that could hinder gauging and recognition of mild, initial, and earlier deformities. Thus, the 3D DMs could represent a more complete, multiplanar and accurate assessment that may provide early identification of previously underappreciated tibiotalar joint changes in early stages of PCFD. Additional longitudinal and prospective studies with different severities of PCFD would help to confirm these possibilities. It is also our hope that through objective quantification using 3D DM and CM we will be able to identify earlier the influences of PCFD in the ankle and syndesmotic joints, potentially preventing late complications such as deltoid ligament insufficiency, syndesmotic instability as well as ankle arthritis. Our findings of significantly decreased articular coverage in the anterior aspects of both the medial and lateral gutters of PCFD patients also support the relatively plantarflexed and possibly anteriorly translated position of the talus in relation to the distal tibia, highlighting the accuracy and potential clinical applicability of 3D CMs as radiologic markers of early changes in PCFD.

The possible clinical applicability of 3D DMs and CMs as earlier WBCT-based radiologic markers of PCFD is supported by considering the results of a recent study. Conti et al. demonstrated that improvements in talar plantarflexion as measured by Meary’s angle (axis of the talus relative to the first metatarsal) following PCFD reconstructive surgery were associated with significant improvements in patient reported outcomes indicating that some aspects of plantarflexion are associated with pain generation^48,49^. However, the correlations seen were relatively weak (r =  − 0.29) and based on a single 2D angular measure of a complex 3D correction. Thus, the regional 3D measures developed herein may provide a foundation for more objective and direct detection of early and subtle malpositioning of the ankle and syndesmotic joints in PCFD patients. They have the potential to extract meaningful information beyond simple plantarflexion performing localized assessments of subregional impingement and widening that may be used to identify more direct correlates of pain generation. Such detailed assessments could enhance precision and enable novel objective analyses of deformity correction following conservative or surgical treatment.

There are several limitations to this study. First, controls were primarily selected from a group of patients with contralateral foot and ankle injuries and deformities. Therefore, subtle asymmetries resulting from antalgic stance may confound our results. We acknowledge that a direct matching process with healthy normal volunteer controls would improve the quality of data collected. However, we believe it would be unlikely to have a substantial impact on DM, CM, or volumetric measurement findings. Second, the volumetric measurements may be impacted by subject size. Our study wanted to enable comparison to syndesmotic injury literature and therefore measured at 1, 3, 5, and 10 cm from the from the tibiotalar joint as reported previously^35,50^. Future studies should optimally seek to normalize these measures to leg length or subject height to account for variability in size. Third, only stage I flexible PCFD patients were evaluated. Future work should seek to study the full range of PCFD including stages I and II, as well as to consider the possible specific findings in the ankle and syndesmotic joints for the different classes of the deformity (A, B, C, D, and E). The lack of formal assessment of deformity severity measurements such as FAO, PTS markers such as subtalar joint subluxation, and presence of sinus and/or subfibular impingement also limit the interpretation of our results. Finally, although a formal power analysis was performed demonstrating sufficient strength, our sample could be underpowered to demonstrate population normality values. However, given the significant findings observed mainly on coverage maps for the tibiotalar joint and ankle gutters, we believe the size of our cohort was robust enough to achieve our objective of examining the utility of CMs as radiologic markers of PCFD.

In conclusion, in this study with PCFD patients and controls, we utilized 3D WBCT distance and coverage maps as well as volumetric measurements to assess joint interaction within the ankle and distal tibiofibular syndesmotic joints. We observed significant decreases in joint coverage of the anterior aspect of the tibiotalar joint, medial and lateral ankle gutters, consistent with early plantarflexion of the talus within the ankle mortise in PCFD patients. Contrary to our hypothesis, syndesmotic volumes were not different when comparing PCFD patients and controls, and the averaged syndesmotic incisura distances were actually significantly smaller in PCFD patients than in controls. These findings, consistent with an absence of syndesmotic widening in PCFD patients, could be explained by talar plantarflexion and resultant internal rotation and medial translation of the fibula. In the presence of peritalar subluxation and syndesmotic stressors like sinus tarsi and particularly subfibular impingement, they may provide important contextual clues about staging and potentially imminent progression. It is our hope that the novel 3D WBCT distance and coverage mapping assessment can enable this kind of early and accurate quantification of the initial and progressive changes in tibiotalar and tibiofibular positioning as well as assisting with treatment decision-making. Further studies are needed to confirm these findings in a prospective and more complete cohort, representing the myriad of different deformities and stages for PCFD, as well as assessing the correlation of these WBCT deformity markers with patient outcomes.

## Data Availability

According to the ICMJE data sharing police, core records will be shared through Endnote Data and available upon request. Requests should be addressed to the corresponding author.

## References

[1] Myerson MS (2020). Classification and nomenclature: Progressive collapsing foot deformity. Foot Ankle Int..

[2] de Cesar Netto C, Deland JT, Ellis SJ (2020). Guest editorial: Expert consensus on adult-acquired flatfoot deformity. Foot Ankle Int..

[3] Deland JT (2008). Adult-acquired flatfoot deformity. J. Am. Acad. Orthop. Surg..

[4] Barg A (2018). Weightbearing computed tomography of the foot and ankle: Emerging technology topical review. Foot Ankle Int..

[5] Godoy-Santos AL, Cesar CN, Weight-Bearing Ct International Study (2018). Weight-bearing computed tomography of the foot and ankle: An update and future directions. Acta Ortop. Bras..

[6] Lintz F (2018). Weight-bearing cone beam CT scans in the foot and ankle. EFORT Open Rev..

[7] de Cesar Netto C (2020). Consensus for the use of weightbearing CT in the assessment of progressive collapsing foot deformity. Foot Ankle Int..

[8] Ananthakrisnan D, Ching R, Tencer A, Hansen ST, Sangeorzan BJ (1999). Subluxation of the talocalcaneal joint in adults who have symptomatic flatfoot. J. Bone Jt. Surg. Am..

[9] Greisberg J, Hansen ST, Sangeorzan B (2003). Deformity and degeneration in the hindfoot and midfoot joints of the adult acquired flatfoot. Foot Ankle Int..

[10] Malakoutikhah H, Madenci E, Latt LD (2022). The impact of ligament tears on joint contact mechanics in progressive collapsing foot deformity: A finite element study. Clin. Biomech. (Bristol, Avon).

[11] Auch E (2021). Distal Tibiofibular syndesmotic widening in progressive collapsing foot deformity. Foot Ankle Int..

[12] Malicky ES (2002). Talocalcaneal and subfibular impingement in symptomatic flatfoot in adults. J. Bone Jt. Surg. Am..

[13] Ellis SJ (2010). Assessment of lateral hindfoot pain in acquired flatfoot deformity using weightbearing multiplanar imaging. Foot Ankle Int..

[14] Apostle KL, Coleman NW, Sangeorzan BJ (2014). Subtalar joint axis in patients with symptomatic peritalar subluxation compared to normal controls. Foot Ankle Int..

[15] Probasco W (2015). Assessment of coronal plane subtalar joint alignment in peritalar subluxation via weight-bearing multiplanar imaging. Foot Ankle Int..

[16] Cody EA, Williamson ER, Burket JC, Deland JT, Ellis SJ (2016). Correlation of talar anatomy and subtalar joint alignment on weightbearing computed tomography with radiographic flatfoot parameters. Foot Ankle Int..

[17] de Cesar Netto C (2019). Subluxation of the middle facet of the subtalar joint as a marker of peritalar subluxation in adult acquired flatfoot deformity: A case-control study. J. Bone Jt. Surg. Am..

[18] de Cesar Netto C (2020). Combined weightbearing CT and MRI assessment of flexible progressive collapsing foot deformity. Foot Ankle Surg..

[19] de Cesar Netto C (2020). Assessment of posterior and middle facet subluxation of the subtalar joint in progressive flatfoot deformity. Foot Ankle Int..

[20] Otero JE (2015). There is poor reliability of Bohler’s angle and the crucial angle of Gissane in assessing displaced intra-articular calcaneal fractures. Foot Ankle Surg..

[21] Colin F, Horn Lang T, Zwicky L, Hintermann B, Knupp M (2014). Subtalar joint configuration on weightbearing CT scan. Foot Ankle Int..

[22] Siegler S (2018). Analysis of surface-to-surface distance mapping during three-dimensional motion at the ankle and subtalar joints. J. Biomech..

[23] Lintz F (2020). Distance mapping of the foot and ankle joints using weightbearing CT: The cavovarus configuration. Foot Ankle Surg..

[24] Dibbern KN (2021). Three-dimensional distance and coverage maps in the assessment of peritalar subluxation in progressive collapsing foot deformity. Foot Ankle Int..

[25] Behrens A (2022). Coverage maps demonstrate 3D Chopart joint subluxation in weightbearing CT of progressive collapsing foot deformity. Sci. Rep..

[26] Lintz F (2017). 3D Biometrics for hindfoot alignment using weightbearing CT. Foot Ankle Int..

[27] Zhang JZ, Lintz F, Bernasconi A, Weight Bearing CTISG, Zhang S (2019). 3D biometrics for hindfoot alignment using weightbearing computed tomography. Foot Ankle Int..

[28] Day MA (2020). Correlation of 3D joint space width from weightbearing CT with outcomes after intra-articular calcaneal fracture. Foot Ankle Int..

[29] Zhang YJ (2019). Correlation between three-dimensional medial longitudinal arch joint complex mobility and medial arch angle in stage II posterior tibial tendon dysfunction. Foot Ankle Surg..

[30] Yoshioka N (2016). Weight-bearing three-dimensional computed tomography analysis of the forefoot in patients with flatfoot deformity. J. Orthop. Sci..

[31] Maceira E, Monteagudo M (2015). Subtalar anatomy and mechanics. Foot Ankle Clin..

[32] Kido M (2013). Load response of the medial longitudinal arch in patients with flatfoot deformity: in vivo 3D study. Clin. Biomech. (Bristol, Avon).

[33] Jackson LT, Aubin PM, Cowley MS, Sangeorzan BJ, Ledoux WR (2011). A robotic cadaveric flatfoot analysis of stance phase. J. Biomech. Eng..

[34] Ashkani Esfahani S (2021). Volume measurements on weightbearing computed tomography can detect subtle syndesmotic instability. J. Orthop. Res..

[35] Bhimani R (2020). Utility of volumetric measurement via weight-bearing computed tomography scan to diagnose syndesmotic instability. Foot Ankle Int..

[36] Myerson MS (1997). Adult acquired flatfoot deformity: Treatment of dysfunction of the posterior tibial tendon. Instr. Course Lect..

[37] Bluman EM, Title CI, Myerson MS (2007). Posterior tibial tendon rupture: A refined classification system. Foot Ankle Clin..

[38] Lalevée M (2021). Prevalence and pattern of lateral impingements in the progressive collapsing foot deformity. Arch. Orthop. Trauma Surg..

[39] Jeng CL, Rutherford T, Hull MG, Cerrato RA, Campbell JT (2019). Assessment of bony subfibular impingement in flatfoot patients using weight-bearing CT scans. Foot Ankle Int..

[40] Beumer A (2006). Effects of ligament sectioning on the kinematics of the distal tibiofibular syndesmosis: A radiostereometric study of 10 cadaveric specimens based on presumed trauma mechanisms with suggestions for treatment. Acta Orthop..

[41] Beumer A (2003). Kinematics of the distal tibiofibular syndesmosis: Radiostereometry in 11 normal ankles. Acta Orthop. Scand..

[42] Beumer A (2004). Radiographic measurement of the distal tibiofibular syndesmosis has limited use. Clin. Orthop. Relat. Res..

[43] Colin F, Zwicky L, Barg A, Hintermann B (2013). Peritalar instability after tibiotalar fusion for valgus unstable ankle in stage IV adult acquired flatfoot deformity: Case series. Foot Ankle Int..

[44] Smith JT, Bluman EM (2012). Update on stage IV acquired adult flatfoot disorder: when the deltoid ligament becomes dysfunctional. Foot Ankle Clin..

[45] Chadha H, Pomeroy G, Manoli A (1997). Radiologic signs of unilateral pes planus. Foot Ankle Int..

[46] Dyal CM, Feder J, Deland JT, Thompson FM (1997). Pes planus in patients with posterior tibial tendon insufficiency: Asymptomatic versus symptomatic foot. Foot Ankle Int..

[47] de Cesar Netto C (2017). Flexible adult acquired flatfoot deformity: Comparison between weight-bearing and non-weight-bearing measurements using cone-beam computed tomography. J. Bone Jt. Surg. Am..

[48] Conti MS, Garfinkel JH, Kunas GC, Deland JT, Ellis SJ (2019). Postoperative medial cuneiform position correlation with patient-reported outcomes following cotton osteotomy for reconstruction of the stage II adult-acquired flatfoot deformity. Foot Ankle Int..

[49] Coughlin MJ, Kaz A (2009). Correlation of Harris mats, physical exam, pictures, and radiographic measurements in adult flatfoot deformity. Foot Ankle Int..

[50] Ashkani Esfahani S (2022). Volume measurements on weightbearing computed tomography can detect subtle syndesmotic instability. J. Orthop. Res..

